# CONNECTING THREADS: SOCIAL PARTICIPATION’S ROLE IN REDUCING LONELINESS AMONG OLDER ADULTS

**DOI:** 10.1093/geroni/igae098.3227

**Published:** 2024-12-31

**Authors:** Nahyun Kim, Keiko Katagiri

**Affiliations:** Kobe University, Kobe, Hyogo, Japan; Kobe University, Kobe, Hyogo, Japan

## Abstract

Social participation among older adults occurs within the context of local community networks, suggesting that engaging in such activities can expand neighborhood networks, enhancing social connections and fostering a sense of belonging. Active social participation serves to both bridge and bond social networks by expanding them to diverse communities and reinforcing close-knit within groups. Despite the acknowledged advantages of social participation in alleviating loneliness, research gaps persist regarding its specific function and effects on loneliness. This study explores the effects of social participation on loneliness, particularly through the lens of bridging new communities and reinforcing internal group bonds, focusing on relationships with weak neighborhood ties and their impact on reducing loneliness. An online survey was conducted with Japanese individuals aged 60–79 years (n=1000), focusing on 570 respondents who were actively involved in social participation. The survey collected data on variables such as bridging to new communities, internal group bonds, the size of weak neighborhood ties (greetings, casual conversations), and loneliness. A path analysis revealed that strong internal group bonds were negatively related to loneliness, highlighting the importance of deep, supportive relationships within groups. Although direct links between the extent of bridging to new communities and immediate loneliness reduction were not found, an indirect positive effect through the expansion of neighborhood networks was identified, associated with decreased loneliness. This highlights the dual benefits of social participation: fostering close-knit group connections and expanding community engagement through weak ties; both are crucial for reducing loneliness and enhancing well-being in older adults.

